# Factors Associated with Non-typhoidal Salmonella Bacteremia versus Typhoidal Salmonella Bacteremia in Patients Presenting for Care in an Urban Diarrheal Disease Hospital in Bangladesh

**DOI:** 10.1371/journal.pntd.0004066

**Published:** 2015-09-11

**Authors:** K. M. Shahunja, Daniel T. Leung, Tahmeed Ahmed, Pradip Kumar Bardhan, Dilruba Ahmed, Firdausi Qadri, Edward T. Ryan, Mohammod Jobayer Chisti

**Affiliations:** 1 Centre for Nutrition and Food Security (CNFS), International Centre for Diarrhoeal Disease Research, Bangladesh (ICDDR,B), Dhaka, Bangladesh; 2 Centre for Vaccine Sciences, International Centre for Diarrhoeal Disease Research, Bangladesh (ICDDR,B), Dhaka, Bangladesh; 3 Division of Infectious Disease, Massachusetts General Hospital, Boston, Massachusetts, United States of America; 4 Department of Medicine, Harvard Medical School, Boston, Massachusetts, United States of America; 5 Dhaka Hospital, International Centre for Diarrhoeal Disease Research, Bangladesh (ICDDR,B), Dhaka, Bangladesh; University of California San Diego School of Medicine, UNITED STATES

## Abstract

**Background:**

Non-typhoidal *Salmonella* (NTS) and *Salmonella enterica* serovar Typhi bacteremia are the causes of significant morbidity and mortality worldwide. There is a paucity of data regarding NTS bacteremia in South Asia, a region with a high incidence of typhoidal bacteremia. We sought to determine clinical predictors and outcomes associated with NTS bacteremia compared with typhoidal bacteremia.

**Methodology:**

We performed a retrospective age-matched case-control study of patients admitted to the Dhaka Hospital of the International Centre for Diarrhoeal Disease Research, Bangladesh, between February 2009 and March 2013. We compared demographic, clinical, microbiological, and outcome variables of NTS bacteremic patients with age-matched *S*. Typhi bacteremic patients, and a separate comparison of patients with NTS bacteremia and patients with NTS gastroenteritis.

**Principal Findings:**

Of 20 patients with NTS bacteremia, 5 died (25% case fatality), compared to none of 60 age-matched cases of *S*. Typhi bacteremia. In univariate analysis, we found that compared with *S*. Typhi bacteremia, cases of NTS bacteremia had more severe acute malnutrition (SAM) in children under five years of age, less often presented with a duration of fever ≥ 5 days, and were more likely to have co-morbidities on admission such as pneumonia and clinical signs of sepsis (p<0.05 in all cases). In multivariable logistic regression, SAM, clinical sepsis, and pneumonia were independent risk factors for NTS bacteremia compared with *S*. Typhi bacteremia (p<0.05 in all cases). Notably, we found marked differences in antibiotic susceptibilities, including NTS strains resistant to antibiotics commonly used for empiric therapy of patients suspected to have typhoid fever.

**Conclusions/Significance:**

Diarrheal patients with NTS bacteremia more often presented with co-morbidities and had a higher case fatality rate compared to those with typhoidal bacteremia. Clinicians in regions where both typhoid and NTS bacteremia are prevalent need to be vigilant about the possibility of both entities, especially given notable differences in antibiotic susceptibility patterns.

## Author Summary

*Salmonella* are a group of bacteria that cause illnesses and death worldwide. There are two types of *Salmonella*–Typhi and non-typhoidal (NTS). In humans, the majority of illnesses caused by NTS are related to gastro-intestinal problems, though uncommonly, it also invades the bloodstream. On the other hand, typhoid fever caused by *Salmonella* typhi commonly invades the bloodstream. Since the treatment of the two types may differ, we wanted to compare the risk factors for each. We studied patients who had NTS or Typhi isolated from blood in a diarrheal hospital in Bangladesh. We observed that patients with NTS bloodstream infection frequently presented with severe malnutrition, clinically diagnosed sepsis and pneumonia compared to those with typhoid fever. We also found that NTS and Typhi differed in what antibiotics they were sensitive to. These observations may help our clinicians to initiate aggressive treatment from the very beginning of the illness in children with NTS bacteremia in order to attain better outcomes.

## Introduction

Non-typhoidal *Salmonella* (NTS) are a group of Gram negative bacteria known to cause disease in both animals and humans worldwide. They include all *Salmonella* enterica *spp*. except for *S*. *enterica* serovar Typhi, Paratyphi A, Paratyphi B, and Paratyphi C. In humans, NTS are responsible for an estimated 94 million cases of gastroenteritis each year globally, causing upwards of 150,000 deaths [1]. NTS is also associated with systemically invasive disease and bacteremia, especially in immunocompromised hosts [2]. In sub-Saharan Africa, NTS is a common cause of bacteremia in both adults and children, especially in areas of high HIV and malaria prevalence [2]. In contrast, the burden of invasive NTS disease in many areas of Asia is thought to be much less than that of sub-Saharan Africa. One multicenter community-based fever surveillance study detected only 6 cases of invasive NTS from over 20,000 blood cultures [3]. Despite this, rates of NTS bacteremia are increasing in Asia, most notably in HIV positive populations [4–6]. Due to the low incidence of disease, there is a paucity of data regarding the clinical presentation, risk factors, resistance patterns, and outcomes for NTS bacteremia in Asia, particularly in areas of low HIV prevalence such as Bangladesh. Given the high incidence of *Salmonella enterica* serovar Typhi infection, a cause of typhoid fever, in this region, comparison and contrast of NTS bacteremia with *S*. Typhi bacteremia may be clinically important, especially with regards to empiric therapy. Also, comparing invasive with non-invasive NTS disease may provide important data for understanding disease pathogenesis.

The aim of this study was to characterize the demographics, clinical presentation, resistance patterns and clinical outcomes of patients admitted to a medical facility in a large urban center in Bangladesh with NTS bacteremia, and to compare them to those associated with *S*. Typhi bacteremia. A secondary objective was to compare cases of NTS bacteremia with those of NTS gastroenteritis.

## Materials and Methods

### Ethical statement

This study was approved by the Research Review and the Ethical Review Committees of the icddr,b and the Institutional Review Board of Massachusetts General Hospital.

### Study setting and population

We retrospectively extracted data from the electronic charting system of the icddr,b Dhaka Hospital, which is a hospital providing care free of charge to approximately 120,000 patients per year, most of whom present with diarrhea. The country of Bangladesh has a HIV prevalence of <0.1% [7], and malaria is not known to occur in the city of Dhaka.

We identified patients admitted to the icddr,b from February 2009 to March 2013 who had *Salmonella* spp. isolated from either blood or stool cultures. We identified all patients with NTS bacteremia (blood culture positive for *Salmonella spp*. other than *S*. Typhi and *S*. Paratyphi), and as a control group, selected age-matched patients with *S*. Typhi bacteremia at a 3:1 ratio. Previous studies from Nepal, Indonesia, and Bangladesh have demonstrated that the clinical presentations of *S*. Typhi and *S*. Paratyphi infection are nearly indistinguishable [8–10]. Thus, to simplify the analysis, we have excluded patients with blood culture positive for S. Paratyphi. In a secondary analysis, we identified patients who had NTS isolated from stool and whose blood culture did not grow any organisms.

### Isolation procedures

Microbiologic culturing of venous blood was performed in patients at the discretion of the attending physician. Blood was seeded directly into BacT/ALERT culture bottles and entered into the BacTAlert 3D system.

Microbiologic culturing of stool was performed as part of a systematic surveillance of every 50^th^ admitted patient, and also for inpatients at the discretion of the attending physician[11]. Stool was cultured using standard isolation methodology from a single, fresh stool specimen collected from the patient [12].

Antibiotic susceptibility testing was performed using Disk Diffusion Method. The detailed procedure has been described elsewhere. [13] Susceptibility pattern was interpreted by using CLSI guideline.[14]

### Diagnosis / definitions of co-morbidities

Pneumonia was diagnosed by following the World Health Organization (WHO) criteria for children under 5 years of age [15]; in patients older than 5 years of age, diagnosis of pneumonia was based on the attending physicians’ interpretation of clinical signs and symptoms and admission chest x-ray, if performed.

Clinical sepsis was defined as presence or suspected presence of infection, plus any two of the following: 1) hypo- (≤35.0°C) or hyperthermia (≥38.5°C), 2) abnormal age-adjusted white blood cell (WBC) count or >10% bands, 3) tachycardia (defined as heart rate above the upper normal limit according to age), 4) tachypnea (defined as respiratory rate above the upper normal limit according to age), and 5) abnormal cognition [16].

Acute kidney injury was defined as a creatinine level greater than the upper limit of normal according to age.

Fever was defined as axillary temperature more than 37.8°C.

Diarrhoea is defined as having loose or watery stools at least three times per day, or more frequently than normal for an individual. [17]

Acute diarrhea was defined as passage of three or greater number of abnormally loose or watery stools in the preceding 24 hours [17].

Acute diarrhea was defined as new onset of diarrhea in a person without a history of diarrhea in the past 14 days

Invasive diarrhea was defined as presence of WBC >20/HPF and any number of red blood cells which was detected by routine and microscopic examination of stool.[18]

Persistent diarrhea was defined as an episode of diarrhea, with or without blood, lasting at least 14 days. [17]

Severe acute malnutrition (SAM) was defined following WHO anthropometry as previously described [19].

Abnormal WBC count was defined as a WBC count outside of the reference values according to the age (0 to 1 month: 6000-36000/cmm; 6 month to 3 years: 6000-17500/cmm; 4 to 10 yrs: 5500-14500/cmm).

Hypokalaemia was defined as a serum potassium level below the reference value (3.5–5.3 mmol/L).

Hyponatraemia was defined as a serum sodium level below the reference value (135–146 mmol/L).

Hypocalcemia was defined as a serum calcium level below the reference value (2.12–2.16 mmol/L).

### Data collection and analysis

De-identified clinical and laboratory data were collected and analyzed using Statistical Package for Social Sciences (SPSS), Windows (Version 17.0; Chicago, IL) and Epi Info (Version 1.0.3, USD, Stone Mountain, GA).

#### Statistics

For continuous variables, the student’s t-test (normally distributed data) or the Mann-Whitney U test (for non-parametric data) was used to compare groups. For categorical variables, Fisher’s exact test was used when a cell value of 2/2 table was <5, and for all other cases, Chi Squared test with Yates correction was used. For comparison between NTS and *S*. Typhi bacteremia, to determine independent predictors of NTS bacteremia, we entered age, gender, and other clinical variables with P < 0.05 from the univariate analysis into a logistic regression model. For comparisons between NTS bacteremia and NTS gastroenteritis, a multivariate analysis was not performed due to small sample size, and we adjusted for multiple comparisons using the Holm–Bonferroni correction.

## Results

In the period February 2009 to March 2013, a total of 12,940 blood cultures were collected from patients admitted to the icddr,b Dhaka hospital. Of these, 20 were positive for NTS, 567 for *S*. Typhi, and 64 were for *S*. Paratyphi (all were Paratyphi A). We compared the clinical and microbiological data from the 20 NTS patients to age-matched controls with *S*. Typhi bacteremia in a 1:3 ratio (60 *S*. Typhi patients). We also identified 27 patients from whom NTS was isolated from stool and not blood (24 blood culture negative, 3 without blood cultures checked). We observed that patients with NTS bacteremia had a significantly higher case fatality rate compared to those with *S*. Typhi bacteremia (25% vs. 0%, p<0.001). The age and causes of death in the five who died with NTS bacteremia are displayed in Table 1.

**Table 1:** Characteristics of NTS bacteremia cases who expired in hospital.

Sl No.	Age	Sex	Length of hospital stay	Was a susceptible antibiotic used?	Presenting symptom / signs
1	70 yrs	F	1	No	Invasive diarrhea
2	25 days	F	3	Yes	Acute watery diarrhea,
3	7 yrs	F	4	Yes	Persistent diarrhea, severe pneumonia,
4	40 yrs	F	2	Yes	Invasive diarrhea, Acute kidney injury
5	7 days	M	2	Yes	Invasive diarrhea, meningitis

We compared the clinical and demographic characteristics of patients with NTS bacteremia with age-matched controls with *S*. Typhi bacteremia. We found that cases of NTS bacteremia were more likely to have SAM, concurrent pneumonia, and clinical sepsis, and less likely to have fever of 5 days or more than those with *S*. Typhi (Table 2). In logistic regression analysis, after adjusting for potential confounders, SAM, pneumonia, and clinical sepsis were independent risk factors for NTS bacteremia (Table 3).

**Table 2:** Demographic, clinical and laboratory characteristics of NTS bacteremia vs *Salmonella* typhi bacteremia.

Variables	NTS bacteremia	*Salmonella* typhi bacteremia	OR	95% CI	p value
	(n = 20)	(n = 60)			
***Demographic and clinical characteristics***					
Age in months (Median, IQR)	14 (3, 540)	15 (10, 420)			0.692
Male Sex	9 (45)	31 (52)	0.77	0.25–2.37	0.605
For <5 years of age, SAM	8/12 (67)	10/36 (28)	5.2	1.07–27.13	0.035
Drinks unboiled supply water	4 (20)	31 (52)	0.23	0.06–0.87	0.026
Duration of diarrhea for 3 days or more	6 (30)	27/56 (48)	0.46	0.13–1.53	0.25
Fever on admission	19 (95)	58 (97)	0.66	0.04–19.39	1.00
Duration of fever for ≥ 5 days	5 (25)	41 (68)	0.12	0.03–0.44	<0.001
Severe dehydration	5/19 (26)	7/56 (12)	2.5	0.57–10.79	0.166
Presence of acute diarrhea	18/19 (95)	55/56 (98)	0.33	0.01–12.74	0.445
Presence of persistent diarrhea / chronic diarrhea	1/19 (5)	1/56 (2)	3.06	0.0–119.2	0.445
Co-morbidity: pneumonia	8 (40)	5 (8)	7.33	1.75–32.2	<0.001
Co-morbidity: clinical Sepsis	9 (45)	2 (3)	23.73	3.9–186	<0.001
***Laboratory characteristics***						
Stool microscopy: Invasive features	3/9 (33)	9/27 (33)	1.0	0.15–6.27	1.0
Hypokalaemia*	4/18 (22)	26/40 (65)	0.15	0.03–0.64	0.006
Hyponatremia*	10/18 (56)	31/40 (77)	0.36	0.09–1.39	0.165
Hypocalcemia*	2/10 (20)	5/6 (83)	0.05	0.00–1.01	0.034
Anaemia on the basis of admission hematocrit (%)	10/17 (59)	44/54 (81)	0.32	0.08–1.24	0.056
Increased creatinine level	12/16 (75)	4/29 (14)	18.75	3.27–127	<0.001
Abnormal WBC count on admission	10/18 (56)	7/54 (13)	8.39	2.13–34.75	<0.001
***Outcome***					
Total hospital stay for discharged alive (days, Median, IQR)	4 (1, 7)	6 (4, 8)			0.016
Death in hospital	5 (25)	0 (0)			<0.001

Figures represent n (%), unless specified. OR: odds ratio. CI: confidence interval, SD: Standard deviation, IQR: Intra quartile range, SAM: severe acute malnutrition, AWD: acute watery diarrhea, AKI: acute kidney injury

**Table 3:** Logistic regression: NTS bacteremia vs. *Salmonella* typhi bacteremia.

Variables	OR	95% CI	p value
For <5 years of age, SAM	22.4	1.7–297.2	0.018
Co-morbidity: clinical sepsis	139.1	5.0–3867.9	0.004
Co-morbidity: pneumonia	16.2	1.4–192.4	0.027
Male gender	3.13	0.32–30.71	0.327
Duration of fever for 5 days or more	2.28	0.19–26.52	0.51
Duration of diarrhea for 3 days or more	0.52	0.62–4.45	0.56

When examining laboratory characteristics on admission, we found that age-matched patients with *S*. Typhi bacteremia were more likely to have hypokalemia, hypocalcemia, and a lower hematocrit in comparison to patients with NTS bacteremia. NTS bacteremic patients were more likely to present with increased creatinine level, and an abnormal (high or low count in age-specific range) white blood count (WBC)

We observed that the susceptibility patterns to conventional antibiotics differed between NTS and *S*. Typhi isolates (Table 4). We found that some strains of NTS had full or intermediate resistance to antibiotics used in the empiric treatment of typhoid fever, such as ceftriaxone and azithromycin.

**Table 4:** Sensitivity pattern of NTS versus typhoidal *Salmonella* spp. recovered from blood.

Antibiotics	Susceptibilities	NTS	*Salmonella* typhi
		(n = 20)	(n = 60)
Ceftriaxone	S	19 (95)	60 (100)
	I	0	0
	R	1 (5)	0
Azithromycin	S	13/15 (87)	47/47 (100)
	I	2/15 (13)	0
	R	0	0
Ampicillin	S	18 (90)	40 (67)
	I	0	0
	R	2 (10)	20 (33)
Chloramphenicol	S	20 (100)	44 (73)
	I	0	0
	R	0	16 (27)
Cotrimoxazole (Trimethoprim + sulfamethoxazole)	S	18 (90)	44 (73)
	I	0	0
	R	2 (10)	16 (27)
Ciprofloxacin	S	11 (55)	4 (7)
	I	9 (45)	56 (93)
	R	0	0

S: susceptible, I: intermediate susceptible, R: resistant

We also examined the differences in clinical characteristics between patients with NTS bacteremia and those who had diarrhea and NTS isolated from stool but not blood. We found that patients with NTS bacteremia were more likely to have a history of drinking unsafe water, to present with fever, to have concurrent pneumonia, and clinical sepsis. However, the significant statistical differences were lost after Holm-Bonferroni correction (Table 5). The serogroups of NTS isolated are shown in Table 6. We found no significant difference between invasive and non-invasive strains with regard to serogroup.

**Table 5:** Clinical and laboratory characteristics of patients with NTS bacteremia versus NTS gastroenteritis.

Variables	NTS bacteremia	NTS gastroenteritis	OR	95% CI	p value	Corrected p value
	(n = 20)	(n = 27)				
***Demographic and clinical characteristics***						
Age in months (Median, IQR)	14 (3, 540)	264 (60, 540)			0.077	0.77
Male sex	9 (45)	14 (52)	0.76	0.20–2.83	0.642	1
For <5, SAM	8/12 (67)	2/6 (33)	4	0.35–55.62	0.321	1
Unboiled supply water for drinking	4/11 (36)	15/19 (79)	0.15	0.02–1.02	0.05	0.55
Duration of diarrhea for 3 days or more	6 (30)	6 (22)	1.50	0.33–6.79	0.545	1
Presence of acute diarrhea	18/19 (95)	27 (100)			0.413	1
Presence of persistent diarrhea / chronic diarrhea	1/19 (5)	0 (0)			0.41	1
Severe dehydration	5/19 (26)	10 (37)	0.57	0.13–2.40	0.58	1
Fever on admission	19 (95)	18 (67)	9.50	1.02–220	0.029	0.377
Duration of fever for 5 days or more	5 (20)	4/20 (20)	1.33	0.24–7.53	1.00	1
Co-morbidity: pneumonia	8 (20)	2 (7)	8.33	1.30–67.89	0.01	0.14
Co-morbidity: Clinical Sepsis	9 (45)	4 (15)	4.7	1–23.76	0.05	0.60
***Laboratory characteristics***						
Stool microscopy: invasive features	3/9 (33)	13 (48)	0.54	0.08–3.24	0.7	1
Features of AKI	12/16 (75)	8/19 (42)	4.13	0.79–23.22	0.11	0.99
Abnormal WBC count on admission	10/18 (56)	9/26 (35)	2.36	0.58–9.81	0.284	1
Anaemia on the basis of admission hematocrit (%)	10/17 (59)	11/25 (44)	1.82	0.44–7.67	0	1
Duration of diarrhea (days) before admission (median, IQR)	2 (1,3)	1 (1,2)			0.113	1
***Outcome***						
Total hospital stay (days) in those discharged alive (median, IQR)	4 (1, 7)	3 (3,4)			0.915	
Death in hospital	5 (25)	0 (0)			0.01	

Figures represent n (%), unless specified. OR: odds ratio. CI: confidence interval, SD: Standard deviation, IQR: Intra quartile range, SAM: severe acute malnutrition, AWD: acute watery diarrhea, ** After Bonferroni-Holm correction.

**Table 6:** Types of NTS isolated from blood or stool.

NTS Type	NTS isolated from blood	NTS isolated from stool
Group B	5 (25)	4 (15)
Group C1	6 (30)	12 (44)
Group C2	1 (5)	3 (11)
Group D	6 (30)	3 (11)
Group E	0	1 (4)
Group G	1 (5)	0
Others	1 (5)	4 (15)

## Discussion

Invasive NTS infections are a cause of significant morbidity and mortality worldwide, most notably in sub-Saharan Africa where it is associated with HIV, malnutrition, and malaria. Here we report the characteristics of patients presenting with invasive NTS to a diarrheal hospital in Dhaka, Bangladesh, where high rates of malnutrition, but not HIV or malaria, are present. We found that NTS bacteremia is a rare occurrence, and that compared with patients with *S*. Typhi bacteremia, patients with NTS bacteremia had a higher in-hospital fatality rate, were more likely to be malnourished, and to have concurrent pneumonia and clinical sepsis.

NTS bacteremia is an extremely uncommon finding at our diarrheal hospital in urban Bangladesh. NTS was isolated in less than 0.2% of all blood cultures, and less than 1% of positive blood cultures. On the other hand, *S*. Typhi accounted for 22% (567/2,573) of all positive blood cultures. We observed that 5 of 20 patients with NTS bacteremia died during hospitalization. This high fatality rate is consistent with studies from Sub-Saharan Africa [20], and underscores the importance of this pathogen despite the lower incidence seen in Asia. When we examined the age distribution of patients with NTS bacteremia, including those who died, we found that the many of the cases were infants and the elderly, age groups known to be of highest risk for invasive NTS. However, we also found cases of NTS bacteremia in older children and middle-aged adults, including in two of the five deaths.

A recent systematic review has identified *S*. Typhi as the most common community-acquired blood stream infection in South and Southeast Asia among both adults and children [21]. Previous studies have reported differences in age distribution between individuals with NTS and *S*. Typhi bacteremia [22]. Thus, we examined the factors differentiating NTS and *S*. Typhi bacteremia through an age-matched case control study using both univariate and multivariable regression analysis. We showed that in children under 5 years of age, SAM was more prevalent in patients with NTS bacteremia than those with *S*. Typhi bacteremia, and in multivariate regression analysis, SAM remained an independent predictor for NTS bacteremia. Studies from Africa have also suggested malnutrition to be a risk factor for NTS infection, compared with non-bacteremic hospitalized children [23,24], and compared with non-*Salmonella* bacteremia [25]. We confirm that this risk factor remains when compared to age-matched patients with *S*. Typhi. The reasons for this is unknown, but we suspect that the immunocompromised state of children with SAM may be a greater predisposing factor for NTS than for *S*. Typhi, which has not been associated with any immunodeficient state. Studies from both developed and developing countries have shown NTS to be more often invasive in immunocompromised patients than the otherwise healthy [26], and the role of the IL-17 axis has been attributed to differences between NTS and *S*. Typhi infection [27]. Furthermore, studies of humans with IL-12p40 deficiency have revealed an increased susceptibility for invasive NTS infection [28], suggesting that IL-12 may play a critical role in protection against NTS. Indeed, IL-12 expression is diminished in malnourished children compared to well-nourished controls [29], and may contribute to their susceptibility to invasive NTS infection.

We showed in our logistic regression analysis that NTS bacteremic patients were more likely to present with concurrent pneumonia and clinical signs of sepsis. Previous studies have shown that both children and adults with NTS bacteremia have co-infection of the lower respiratory tract [23,30]. Given the similarity in clinical signs and symptoms of bacterial pneumonia and NTS bacteremia [31], differences in microbial etiology and choice of empiric antibiotics, and the high mortality rate of NTS, care must be taken to consider the presence of NTS bacteremia in cases of lower respiratory tract infection where risk factors of NTS are present.

Previous reports from our institution have shown that drinking unboiled water is a risk factor for typhoid fever [32], but that the type of water source is not a risk factor for NTS infection [33]. In this study, we show that patients with *S*. Typhi bacteremia are more likely to drink unboiled water than those with NTS bacteremia. This may be a reflection of the large role that contaminated food and water plays in the acquisition of typhoid (a human-restricted pathogen), and does not rule out the possibility that NTS is acquired through contaminated food and water.

The higher proportion of NTS patients presenting with clinical signs of sepsis, acute kidney injury, and abnormal WBC count, together with associated mortalities due to septic shock, are all likely a reflection of the higher severity of illness on presentation in NTS bacteremia compared to *S*. Typhi bacteremia. Despite the higher severity of illness, we found that patients with NTS bacteremia are more likely to present with a shorter duration of fever on admission, similar to findings from a study of Tanzanian children in a malaria-endemic region [34]. The reasons for this are unclear, but may include factors such as higher rates of malnutrition among NTS patients and respiratory co-infections as described above. We hypothesize that patients with NTS blood stream infection may progress to severe disease more quickly than those with *S*. Typhi. On the other hand, duration of fever was not an independent predictor based on multivariable regression, and previous studies suggest that NTS and typhoid fever may have similar duration of fever.[35,36] Thus, studies with larger sample sizes are needed to clarify whether severity of illness on presentation is clearly different between the two entities.

Patients presenting with *S*. Typhi bacteremia also had a lower hematocrit than those with NTS. This is in contrast to findings from the Tanzanian study, which did not account for the contribution of age (NTS patients younger) nor malaria co-infection. Hematological changes are common in typhoid fever and bone marrow suppression and hemophagocytosis are considered to be an potential mechanisms for such effects [37]. We hypothesize that the higher proportion of anemia and electrolyte imbalances seen in *S*. Typhi-infected patients may also be indicative of the more insidious nature of typhoid fever and lengthier time prior to presentation to hospital.

The resistance patterns of blood NTS isolates in this report are comparable to previous reports of stool NTS isolates from Bangladesh [33], including high rates of intermediate resistance to ciprofloxacin, and lower rates of resistance to ampicillin, chloramphenicol, and TMP/SMX. However, there were several differences between the antimicrobial sensitivity patterns of isolated NTS and *S*. Typhi strains. Most notably, we found strains of NTS isolated from bacteremic patients that are resistant to ceftriaxone, and two with intermediate resistance to azithromycin. All strains of *S*. Typhi isolated were fully susceptible to the above agents, consistent with previous reports [36,38,39], and ceftriaxone (or oral equivalent) and azithromycin are commonly used for empiric therapy of patients suspected to have typhoid fever. While we did not find significant differences in time-to-appropriate antibiotic between the two groups, the differing susceptibility patterns of NTS and *S*. Typhi may have implications for empiric treatment of children presenting with fever.

Our study has several notable limitations. First, this is a hospital-based study and thus our findings can only be generalized to individuals in the population whose symptoms were severe enough to seek medical care. Also, since the majority of patients presenting to our diarrheal hospital have diarrhea, our findings may be biased towards those who prominently presented with diarrhea. Secondly, this was a retrospective study of a population where not all admitted patients had blood cultures drawn, and thus there is a selection bias towards those who had clinical suspicion for bacteremia. Thirdly, we did not test patients for HIV, though the prevalence of HIV in Bangladesh is low at < 0.1% (7). Fourthly, we only used conventional culture methods to detect *Salmonella* infections, as assays using other methodologies were not routinely available. Fifthly, the numbers of NTS bacteremia cases are low (n = 20), our confidence intervals are quite wide, and larger studies are needed to confirm our findings. We matched by age so that we could compare other features; however, our approach could also have introduced limitations for overall analysis by infecting pathogen. We also focused our analysis of enteric fever to that caused by *S*. Typhi, and did not include enteric fever caused by *S*. Paratyphi A in our analysis, given previous reports demonstrating their similarities in clinical presentation. Lastly, as we had extracted the data from the hospital EMR, we were not able to ascertain pre-admission use of antimicrobials, which may influence the severity and duration of presenting symptoms. Despite these limitations, our study is the largest analysis of NTS bacteremia in South Asia and largely matches findings from sub-Saharan Africa,[22,40] Given the influence of HIV and malaria co-infection on NTS infection in sub-Saharan Africa, we believe that our report is significant and of particular import to those working in Asia.

In conclusion, we have compared clinical and microbiological characteristics of patients with NTS and *S*. Typhi bacteremia at a diarrheal hospital in Bangladesh, and found a higher incidence of comorbidities on presentation and a higher fatality rate among those with NTS bacteremia. These results, combined with a different antibiotic susceptibility profile than *S*. Typhoid isolates, warrant further investigation into the epidemiology of invasive NTS infections in South Asia, and surveillance of resistance patterns of both non-typhoidal and typhoidal *Salmonella* infections in this region.

## Supporting Information

S1 Checklist STROBE Statement—Checklist of items that should be included in reports of cross-sectional studies. (DOC)
