# Supplementary material for: Factors Associated with Non-typhoidal Salmonella Bacteremia versus Typhoidal Salmonella Bacteremia in Patients Presenting for Care in an Urban Diarrheal Disease Hospital in Bangladesh
Source: PLoS Negl Trop Dis. 2015 Sep 11;9(9):e0004066. doi: 10.1371/journal.pntd.0004066 (PMC4567379; doi:10.1371/journal.pntd.0004066)
Supplement: S1 Checklist — (DOC) [file pntd.0004066.s001.doc]

STROBE Statement—Checklist of items that should be included in reports of ***cross-sectional studies***

|  | Item No | Recommendation |
| --- | --- | --- |
| **Title and abstract** | 1 | (*a*) Indicate the study’s design with a commonly used term in the title or the abstract (available on page: 1-2) |
|  |  | (*b*) Provide in the abstract an informative and balanced summary of what was done and what was found (available on page: 2) |
| Introduction | | |
| Background/rationale | 2 | Explain the scientific background and rationale for the investigation being reported (available on page: 4) |
| Objectives | 3 | State specific objectives, including any pre-specified hypotheses (available on page: 4) |
| Methods | | |
| Study design | 4 | Present key elements of study design early in the paper (available on page: 5) |
| Setting | 5 | Describe the setting, locations, and relevant dates, including periods of recruitment, exposure, follow-up, and data collection (available on page: 5) |
| Participants | 6 | (*a*) Give the eligibility criteria, and the sources and methods of selection of participants (available on page: 5) |
| Variables | 7 | Clearly define all outcomes, exposures, predictors, potential confounders, and effect modifiers. Give diagnostic criteria, if applicable (available on page: 5-7 ) |
| Data sources/ measurement | 8* | For each variable of interest, give sources of data and details of methods of assessment (measurement). Describe comparability of assessment methods if there is more than one group (available on page: 7) |
| Bias | 9 | Describe any efforts to address potential sources of bias (available in limitation section on page 11) |
| Study size | 10 | Explain how the study size was arrived at (available on page: 5) |
| Quantitative variables | 11 | Explain how quantitative variables were handled in the analyses. If applicable, describe which groupings were chosen and why (available on page: 7) |
| Statistical methods | 12 | (*a*) Describe all statistical methods, including those used to control for confounding (available on page: 7) |
|  |  | (*b*) Describe any methods used to examine subgroups and interactions (not applicable) |
|  |  | (*c*) Explain how missing data were addressed (not applicable) |
|  |  | (*d*) If applicable, describe analytical methods taking account of sampling strategy (not applicable) |
|  |  | (*e*) Describe any sensitivity analyses (not applicable) |
| Results | | |
| Participants | 13* | (a) Report numbers of individuals at each stage of study—eg numbers potentially eligible, examined for eligibility, confirmed eligible, included in the study, completing follow-up, and analysed (available on page: 7) |
|  |  | (b) Give reasons for non-participation at each stage (not applicable) |
|  |  | (c) Consider use of a flow diagram (not used as the number of sample is straight-forward and has clearly been stated on page 8) |
| Descriptive data | 14* | (a) Give characteristics of study participants (eg demographic, clinical, social) and information on exposures and potential confounders (available on page: 8) |
|  |  | (b) Indicate number of participants with missing data for each variable of interest (not applicable) |
| Outcome data | 15* | Report numbers of outcome events or summary measures (available on page: 8) |
| Main results | 16 | (*a*) Give unadjusted estimates and, if applicable, confounder-adjusted estimates and their precision (eg, 95% confidence interval). Make clear which confounders were adjusted for and why they were included (available on page: 8) |
|  |  | (*b*) Report category boundaries when continuous variables were categorized |
|  |  | (*c*) If relevant, consider translating estimates of relative risk into absolute risk for a meaningful time period bias (not applicable for this retrospective chart analyses)  (*d*) Supporting Information Legends section ( **available on page 16**) |
| Other analyses | 17 | Report other analyses done—eg analyses of subgroups and interactions, and sensitivity analyses bias (not applicable for this retrospective chart analyses) |
| Discussion | | |
| Key results | 18 | Summarise key results with reference to study objectives (available on page: 9-10) |
| Limitations | 19 | Discuss limitations of the study, taking into account sources of potential bias or imprecision. Discuss both direction and magnitude of any potential bias (available on page: 11) |
| Interpretation | 20 | Give a cautious overall interpretation of results considering objectives, limitations, multiplicity of analyses, results from similar studies, and other relevant evidence (available in the discussion section on page 11-12) |
| Generalisability | 21 | Discuss the generalisability (external validity) of the study results (available on page: 12 ) |
| Other information | | |
| Funding | 22 | Give the source of funding and the role of the funders for the present study and, if applicable, for the original study on which the present article is based (available on page: 12) |

*Give information separately for exposed and unexposed groups.

**Note:** An Explanation and Elaboration article discusses each checklist item and gives methodological background and published examples of transparent reporting. The STROBE checklist is best used in conjunction with this article (freely available on the Web sites of PLoS Medicine at http://www.plosmedicine.org/, Annals of Internal Medicine at http://www.annals.org/, and Epidemiology at http://www.epidem.com/). Information on the STROBE Initiative is available at www.strobe-statement.org.
